# Rib Fractures and Death from Deletion of Osteoblast βcatenin in Adult Mice Is Rescued by Corticosteroids

**DOI:** 10.1371/journal.pone.0055757

**Published:** 2013-02-05

**Authors:** JinZhu Duan, Yueh Lee, Corey Jania, Jucheng Gong, Mauricio Rojas, Laurel Burk, Monte Willis, Jonathon Homeister, Stephen Tilley, Janet Rubin, Arjun Deb

**Affiliations:** 1 Department of Medicine, University of North Carolina, Chapel Hill, North Carolina, United States of America; 2 Department of Cell Biology and Physiology, University of North Carolina, Chapel Hill, North Carolina, United States of America; 3 Department of Physics and Astronomy, University of North Carolina, Chapel Hill, North Carolina, United States of America; 4 Department of Radiology, University of North Carolina, Chapel Hill, North Carolina, United States of America; 5 Department of Pathology and Laboratory Medicine, University of North Carolina, Chapel Hill, North Carolina, United States of America; 6 Division of Cardiology, University of North Carolina, Chapel Hill, North Carolina, United States of America; 7 Division of Endocrinology, University of North Carolina, Chapel Hill, North Carolina, United States of America; 8 Division of Pulmonology, University of North Carolina, Chapel Hill, North Carolina, United States of America; 9 Curriculum in Genetics and Molecular Biology, University of North Carolina, Chapel Hill, North Carolina, United States of America; 10 UNC McAllister Heart Institute, University of North Carolina, Chapel Hill, North Carolina, United States of America; 11 Lineberger Comprehensive Cancer Center, University of North Carolina, Chapel Hill, North Carolina, United States of America; INSERM U1059/LBTO, Université Jean Monnet, France

## Abstract

Ribs are primarily made of cortical bone and are necessary for chest expansion and ventilation. Rib fractures represent the most common type of non-traumatic fractures in the elderly yet few studies have focused on the biology of rib fragility. Here, we show that deletion of βcatenin in Col1a2 expressing osteoblasts of adult mice leads to aggressive osteoclastogenesis with increased serum levels of the osteoclastogenic cytokine RANKL, extensive rib resorption, multiple spontaneous rib fractures and chest wall deformities. Within days of osteoblast specific βcatenin deletion, animals die from respiratory failure with a vanishing rib cage that is unable to sustain ventilation. Increased bone resorption is also observed in the vertebrae and femur. Treatment with the bisphosphonate pamidronate delayed but did not prevent death or associated rib fractures. In contrast, administration of the glucocorticoid dexamethasone decreased serum RANKL and slowed osteoclastogenesis. Dexamethasone preserved rib structure, prevented respiratory compromise and strikingly increased survival. Our findings provide a novel model of accelerated osteoclastogenesis, where deletion of osteoblast βcatenin in adults leads to rapid development of destructive rib fractures. We demonstrate the role of βcatenin dependent mechanisms in rib fractures and suggest that glucocorticoids, by suppressing RANKL, may have a role in treating bone loss due to aggressive osteoclastogenesis.

## Introduction

βcatenin plays a critical role in commitment and differentiation of mesenchymal progenitors into different lineages and promotes osteoblast differentiation [1], [2], [3], [4]. Osteoblasts not only form new bone but also control bone resorption by modulating osteoclast formation. Osteoblasts express the osteoclastogenic cytokine RANKL (receptor activator of nuclear factor kappa B ligand) that binds to RANK receptor present on osteoclast progenitors and induces osteoclast formation. RANKL is a critical determinant of osteoclastogenesis and close coupling of osteoblast and osteoclast activity regulates bone mass in the adult mammal [5]. In age related osteoporosis or bone loss related to excessive osteoclastogenesis, the skeleton does not lose bone in a uniform distribution, rather some skeletal sites exhibiting a far greater degree of bone loss compared to other regions. The biological basis of such skeletal site specific osteoclastogenesis remains unclear. βcatenin activity in bone is affected by physical loading of the skeleton and is important for skeletal site specific response to load [6], [7]. Osteoblast βcatenin modulates RANKL activity and osteoclastogenesis [8] but whether site specific modulation of osteoclastogenesis by βcatenin occurs remains unknown.

Although osteoporotic studies concentrate on fractures of the hip and spine, rib fractures represent the most common non-traumatic fractures in the elderly and a large fraction of rib fractures occur in the absence of osteopenia [9], [10]. Rib fractures and deformity leading to cardiopulmonary insufficiency are the leading cause of death in osteogenesis imperfecta [11]. Site specific osteoclastogenesis predominating in ribs also occurs in metastatic cancer and myeloma [12] but few studies have focused on the biology of site specific skeletal fragility or queried a possible contribution of local βcatenin expression in regulating osteoclastogenesis and fractures in ribs. Here, we present a model of βcatenin regulated skeletal site specific osteoclastogenesis with increased RANKL levels, aggressive rib resorption and fractures, and demonstrate the potential use of corticosteroids in suppressing RANKL to interrupt rapid osteoclastogenesis.

## Materials and Methods

### Ethics Statement

This study was carried out in strict accordance with the recommendations in the Guide for the Care and Use of Laboratory Animals of the National Institutes of Health. This protocol was approved by the Institutional Animal Care and Use Committee of the University of North Carolina at Chapel Hill. Analgesics were appropriately used to minimize suffering.

### Generation of *Col1a2CreERT/βcatenin* ^fl/fl^ Mice

*Col1a2CreERT* transgenic mice carry a tamoxifen inducible Cre recombinase element under the control of a promoter sequence of pro α2(I) collagen gene [13]. Cre transgenic mice (C57Bl/6) were crossed with mice having both βcatenin alleles floxed (βcatenin^fl/fl^) (C57Bl/6) to generate mice heterozygous for both alleles. Mice heterozygous for both alleles were backcrossed with βcatenin^fl/fl^ mice to yield *Col1a2CreERT*/βcatenin^fl/fl^ mice. Tamoxifen dissolved in corn oil (10mg/mL) was administered intra-peritoneally daily for 11 days in 8 week old *Col1a2CreERT*/βcatenin^fl/fl^ mice (23 male and 22 female) to delete βcatenin in *Cre* expressing cells. Analysis of survival was performed following completion of 10 days of tamoxifen administration. For lineage reporter analysis, *Col1a2CreERT* mice or *Cola2CreERT/*βcatenin^fl/fl^ mice were crossed to *Rosa26R^lacZ^* transgenic mice to create *Col1a2CreERT/Rosa26R^lacZ^* or *Cola2CreERT/*βcatenin^fl/fl^ */Rosa26R^lacZ^* mice. Pamidronate (5mg/kg) was injected intra-peritoneally 5 days prior to starting tamoxifen injections and continued twice/weekly for the duration of analysis. Dexamethasone phosphate (equivalent to 1mg/kg dexamethasone) was administered subcutaneously thrice/weekly, started concomitantly with tamoxifen, or after stopping tamoxifen and continued for the duration of the analysis. This dose of dexamethasone is consistent with that used in several murine studies investigating the effects of this agent on bone loss and is similar to that used in humans for treating inflammatory states [14], [15], [16].

### Pulmonary Function Testing and Estimation of Oxygen Saturation

Mice were anesthetized with sodium pentobarbital, subjected to tracheostomy and mechanically ventilated with a computer controlled small animal ventilator and pulmonary function testing was done with invasive testing as described [17]. Briefly, mice were paralyzed with pancuronium bromide and airway pressure, volume and flow were measured using a precisely controlled piston during a single inspiration and expiration, with custom designed software (Flexivent, Scireq). Forced oscillation techniques were used to calculate airway impedance. For measurement of peripheral blood oxygen saturation, mice were anesthetized with a 3 minute isoflurane challenge and a mouse-OX (Starr Life Science) oximeter probe was placed on the scruff of the neck to determine the oxygen saturation.

### CT Imaging for Lung and Bone Volumes

Tomographic images were acquired with a field emission x-ray cone beam micro CT following image acquisition techniques as described [18]. Image acquisition was physiologically gated to eliminate motion blur, synchronizing x-ray exposures with the inhalation phase of respiration. Respiration monitoring was performed with a novel contactless fiber optic displacement sensor. The sensor was positioned adjacent to the animal’s rib cage and was able to detect minute changes in chest position during respiration. Each CT scan required approximately 8–15 minutes for completion, dependent upon the subject’s respiration rate. Images were reconstructed into DICOM format for analysis. Bone samples were scanned by SCANCO µCT 40 for image acquisition and assessment of bone density. Region of Interest (ROI) analysis was performed with Image J software. Trabecular bone analysis was performed by drawing an ROI in the center of the vertebral body (no greater than 1/3 of the diameter of the vertebral body) and in the femur (just proximal to the distal femoral metaphysis). This region was found to be most repeated identifiable across the animals. Care was taken to draw the ROIs in consistent anatomic locations with ROI volumes of at least 1000 voxels, across at least 3 slices each as described [18]. Cortical bone volume in the ribs was measured based on an ROI circling the rib on an image in tangential cross section (smallest local diameter). The outside of the ROI encircled the cortex, with the inner medullary portion excluded with a second ROI. Bone volume was then calculated based on percentage of pixels exceeding a consistent threshold defined for each bone type, and all imaging for each bone type was performed under consistent imaging parameters. Image analysis was performed with Image J. *In vivo* pulmonary images were also acquired with a field emission X-ray cone beam micro-CT. 3 D reconstructions created with appropriate software (Volview, www.kitware.com) [19].

### Immunohistochemistry, Xgal Staining and RANKL Assay

For Xgal staining, tissues were harvested and fixed in 4% Paraformaldehyde at 4°C either overnight (whole mount staining) or for 4 hours (cryosection). Subsequently, tissues were decalcified with 14% EDTA for 24 hours. Xgal staining was then done overnight with Xgal staining solution (1 mg/ml Xgal, 5 mM potassium ferrocyanide, and 5 mM potassium ferricyanide, 2 mM MgCl2, 0.01% sodium deoxycholate, 0.02% Nonidet-P40 (NP-40) in PBS) at 37°C. For immunohistochemistry, ribs cryosections were fixed in acetone for 10min at -20°C followed by staining according to the manufacturer’s instructions (Vector Labs ABC kit). Primary antibodies including RANKL antibody (10ug/ml, Petrotech), alkaline phosphatase antibody (5ug/ml, R&D), βgalactosidase antibody (1∶1000, MP Biomedicals), βcatenin antibody (1∶100, Cell signaling technology) and antibodies B220, CD8, F4/80(1∶100, eBioscience) were incubated overnight at 4°C. Biotinylated secondary antibodies and Avidin D FITC or Texas Red (Vector Biolabs) were used as appropriate. A Leica SP2 AOBS upright laser scanning confocal microscope was used to obtain images. A mouse RANKL Elisa kit (R&D) was used to measure peripheral blood plasma RANKL levels.

### Alkaline Phosphatase and TRAP Staining

Commercially available kits were used for determining alkaline phosphatase and TRAP activity (Sigma). For alkaline phosphatase staining, ribs were collected and frozen in O.C.T. compound immediately followed by staining according to the manufacturer’s instructions. For TRAP staining, bone was initially fixed in 4%PFA for 24 hours at 4°C, subjected to decalcification for 36 hours in 14% EDTA at 4°C and then kept in 70% ethanol prior to paraffin embedding. Sections were subsequently deparaffinized and TRAP activity determined by following manufacturer’s instructions.

### Complete Blood Counts and Serum Chemistry

Blood was collected in micro tubes containing EDTA (BD Biosciences) following submandibular venesection in mice. Peripheral blood counts were analyzed by Heska’s animal blood counter. For blood chemistries, plasma was collected using vacuum tubes (BD Biosciences) and samples analyzed with an automated chemical analyzer (Johnson and Johnson VT350).

### Statistics

p values were computed with student’s t test and one way Anova with Bonferroni’s post hoc tests as appropriate. All statistical analysis was completed using Graph Pad Prism software.

## Results

### Deletion of Osteoblast βcatenin Leads to Respiratory Failure and Death from Rib Fractures and a Flail Chest Wall

**Figure 1:**
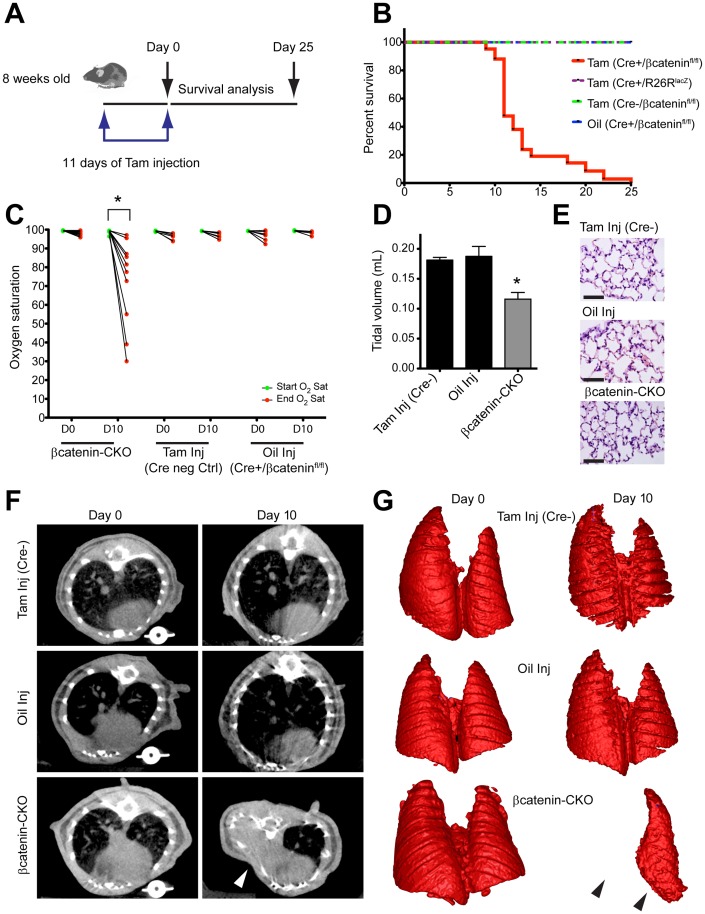
βcatenin CKO mice die within days of βcatenin deletion. (**A**) Strategy for deleting βcatenin in *Col1a2* expressing cells of 8 week old *Col1a2CreERT*/βcatenin^fl/fl^ mice. (**B**) Survival of mice following tamoxifen administration (n = 45 animals for βcatenin-CKO and15 animals for each of the control groups). (**C**) Peripheral blood oxygen saturation of mice pre and post pulmonary challenge with inhaled isoflurane (n = 10 animals in βcatenin-CKO group and 5–6 animals for control groups, *p<0.001) (**D**) Tidal volume (mean±S.E.M.; n = 6 animals/group, *p<0.05 versus control groups) and (**E**) lung histology, 10 days post tamoxifen (**F**) CT scan and (**G**) 3-dimensional lung reconstruction at Day 0 and Day 10 post tamoxifen (n = 6 animals/group). (White arrowheads indicate chest wall deformity and black arrowheads loss of lung volume. Oil injection refers to Cre+/βcatenin^fl/fl^ animals injected with oil and Cre neg control refers to tamoxifen injected βcatenin^fl/fl^ animals). (Scale bar: 100 µm).

While studying the role of βcatenin in regulating lineage and function of cells of mesenchymal origin, we observed that deletion of βcatenin in osteoblasts of adult mice led to rapid development of spontaneous rib fractures, flail chest and death from respiratory insufficiency. βcatenin was deleted in osteoblasts by crossing transgenic mice expressing tamoxifen inducible Cre recombinase driven by the *Col1a2* promoter (*Col1a2CreERT*) with mice having both βcatenin alleles floxed (βcatenin^fl/fl^) [20], [21]. Tamoxifen was administered daily for 11 days to 8 week old *Col1a2CreERT*:βcatenin^fl/fl^ mice to excise βcatenin in *Col1a2* expressing cells (βcatenin-conditional knock out or βcatenin-CKO) **(** **Figure 1A** **)**. Lineage analysis using the *Col1a2CreERT*:*R26R^lacZ^* reporter mice demonstrated that in addition to osteoblasts in bone, smooth muscle cells and fibroblasts in the heart, lung, liver, spleen, skeletal muscle and kidney expressed *Col1a2* **(Figure S1)**. Within several days of the last tamoxifen dose, βcatenin-CKO animals developed labored breathing with median survival of 11 days and 100% lethality by day 25 **(** **Figure 1B** **)**. Not a single death occurred in the vehicle (oil) injected animals or tamoxifen injected βcatenin^fl/fl^ or *Col1a2CreERT:R26R^lacZ^* animals **(** **Figure 1B** **)**.

To determine why deletion of βcatenin in *Col1a2* expressing cells of adult mice led to labored breathing and rapid demise, we subjected the mice to a pulmonary challenge ten days after the last dose of tamoxifen. Following inhalation of low dose isoflurane for 3 minutes, βcatenin-CKO animals exhibited a profound peripheral oxygen desaturation of 26% (98±0.36% vs 72±7%, mean±S.E.M., p<0.001) **(** **Figure 1C** **)**; 20% of the animals died during isoflurane challenge. Mice in the control groups had minimal changes (<5%, p>0.05) in their peripheral oxygen saturation **(** **Figure 1C** **)**. Invasive measurements demonstrated 50% reduction in tidal volume and significantly abnormal ventilatory parameters in the βcatenin-CKO animals **(** **Figure 1D** **and Figure S2)**. Computer assisted tomography (CT) scans and 3D reconstruction of the lungs of βcatenin-CKO animals demonstrated chest wall asymmetry and lung collapse **(** **Figure 1F,G** **)**. However, lung histology did not reveal any abnormalities **(** **Figure 1E** **)**. Cardiac function, blood biochemical tests and peripheral blood counts were also normal except for mild neutropenia **(Table S1, S2, S3 and Figure S3)**. The combination of lung collapse and asymmetry of the chest wall associated with histologically normal lungs suggested defects in the thoracic skeleton as the likely etiology of impaired ventilation and respiratory insufficiency.

### Rapid and Extensive Bone Loss Secondary to Osteoclastogenesis Leads to Rib Fractures

**Figure 2:**
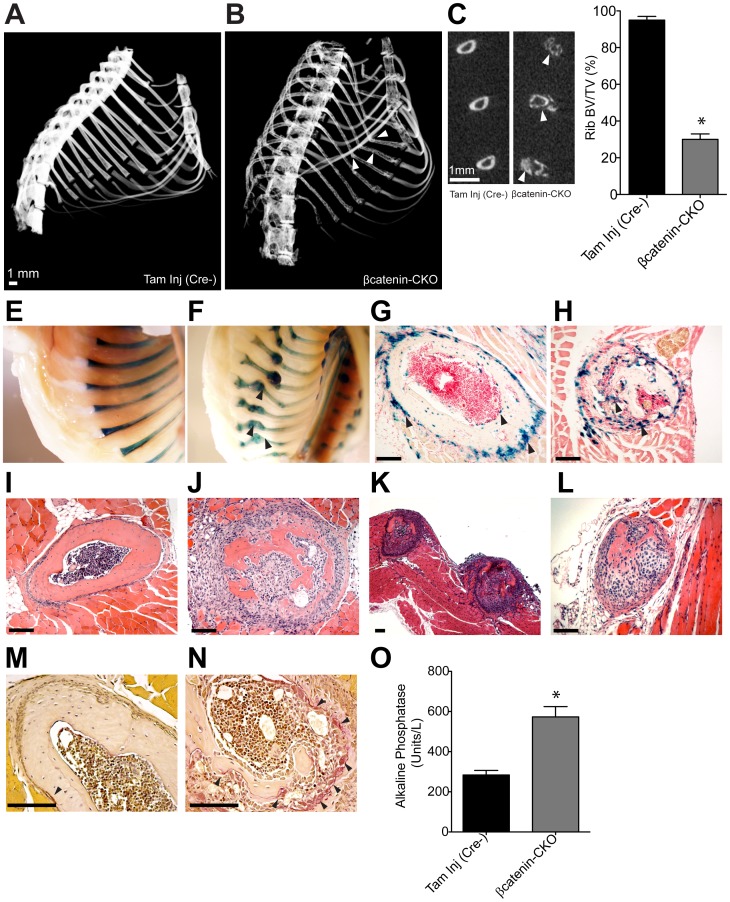
βcatenin-CKO mice exhibit extensive rib resorption from osteoclastogenesis. (**A–C**) Micro CT of the thoracic cage of (**A**) control and (**B**) βcatenin-CKO animals. White arrowheads point to osteopenia and rib deformity. (**C**) Ribs shown in coronal plane with bone destruction (arrowheads) with (**D**) Rib bone volume/total volume, BV/TV) (mean±S.E.M.; n = 6 animals/group, *p<0.0001, BV/TV measurements were made on cortical rib) (**E,F**) Xgal staining *in-situ* of rib cage of (**E**) *Col1a2CreERT* and (**F**) βcatenin-CKO lineage reporter animals with callus and fracture deformities (black arrowheads). (**G,H**) Xgal staining of rib sections of (**G**) *Col1a2CreERT* and (**H**) βcatenin-CKO lineage reporter animals (arrowheads point to Cre expressing cells in blue) (**I–L**) Hematoxylin-eosin staining of rib sections of (**I**) control and (**J–L**) βcatenin-CKO animal showing (**J**) gross rib destruction with cellular infiltrate (**K**) involvement of consecutive ribs (**L**) complete rib resorption (**M,N**) TRAP staining of rib sections with TRAP positive cells (arrowheads) in (**M**) control and (**N**) βcatenin-CKO animals (**O**) Plasma alkaline phosphatase (mean±S.E.M., n = 10 animals/group, *p<0.05). All measurements and staining were done in animals 10 days post completion of tamoxifen. (Scale bar: 100 µm).

To determine potential defects in the chest wall micro CT scans of the entire thoracic cage were performed. Within 10 days of completing tamoxifen dosing, ribs in the βcatenin-CKO animals were overtly osteopenic with deformities due to multiple fractures **(** **Figure 2A,B** **and Video S1, S2)**. High resolution CT scan of the ribs confirmed gross bone destruction with a decrease in rib bone volume (bone volume/total volume, BV/TV) by 66±3% compared to control groups (p<0.0001) (**Figure 2C,D**). To evaluate expression of *Col1a2* in the ribs of the affected animals, βcatenin-CKO animals were crossed with the lineage reporter *R26R^lacZ^* mice to generate βcatenin-CKO lineage reporter animals (*Col1a2CreERT*:βcatenin^fl/fl^:*R26R^lacZ^*). X gal staining of the rib cage *in situ* demonstrated uniform lacZ expression across the surface of the ribs in the *Col1a2* lineage reporter (*Col1a2CreERT*:*R26R^lacZ^*) animals **(** **Figure 2E** **)**. In βcatenin-CKO lineage reporter animals, lacZ expression was intensified in areas of callus formation at multiple fracture sites **(** **Figure 2F** **)**. Xgal staining of rib sections of βcatenin*-CKO*:*R26R^lacZ^* animals demonstrated *Col1a2* expressing cells scattered throughout the area of bony destruction while in control animals they were prominently present in the endosteal and periosteal surfaces as well as in cortical bone **(** **Figure 2G,H** **)**.

Using alkaline phosphatase to identify osteoblasts, we found that *Col1a2* expressing cells in the ribs were cells of osteoblast lineage and that βcatenin protein was not detectable in 80% of these cells ten days post tamoxifen **(Figure S4A,B)**. Hematoxylin-eosin staining of sections of βcatenin-CKO ribs demonstrated extensive destruction of bone associated with a dense cellular infiltrate surrounding the areas of bone loss compared to control mice **(** **Figure 2I,J** **)**. Consecutive ribs were affected and multiple ribs demonstrated areas of near complete rib resorption **(** **Figure 2K,L** **)**. The cells comprising the infiltrate in the region of rib destruction were largely alkaline phosphatase positive, likely representing coupled bone formation in response to bone loss **(Figure S5A,B)**. Masson trichrome staining (identifies collagen) demonstrated significant decrease in rib osteoid **(Figure S6A–C)** and Von Kossa staining (identifies calcium deposits) showed a similar decrease in calcium content of the affected ribs **(Figure S6D–F)**.

We next investigated whether rapid bone loss in the ribs was secondary to osteoclastogenesis. Tartrate resistant acid phosphatase (TRAP) staining to identify osteoclasts showed a significant increase in the number of osteoclasts in βcatenin-CKO ribs **(** **Figure 2M,N** **)**. Blood alkaline phosphatase was significantly elevated by 2 fold in βcatenin-CKO groups, consistent with extensive bone remodeling **(** **Figure 2O** **)**. These data thus suggest that rib destruction was secondary to extensive osteoclast recruitment following βcatenin deletion in rib osteoblasts expressing *Col1a2*.

### βcatenin-CKO Animals Exhibit Less Dramatic Bone Resorption at other Skeletal Sites

**Figure 3:**
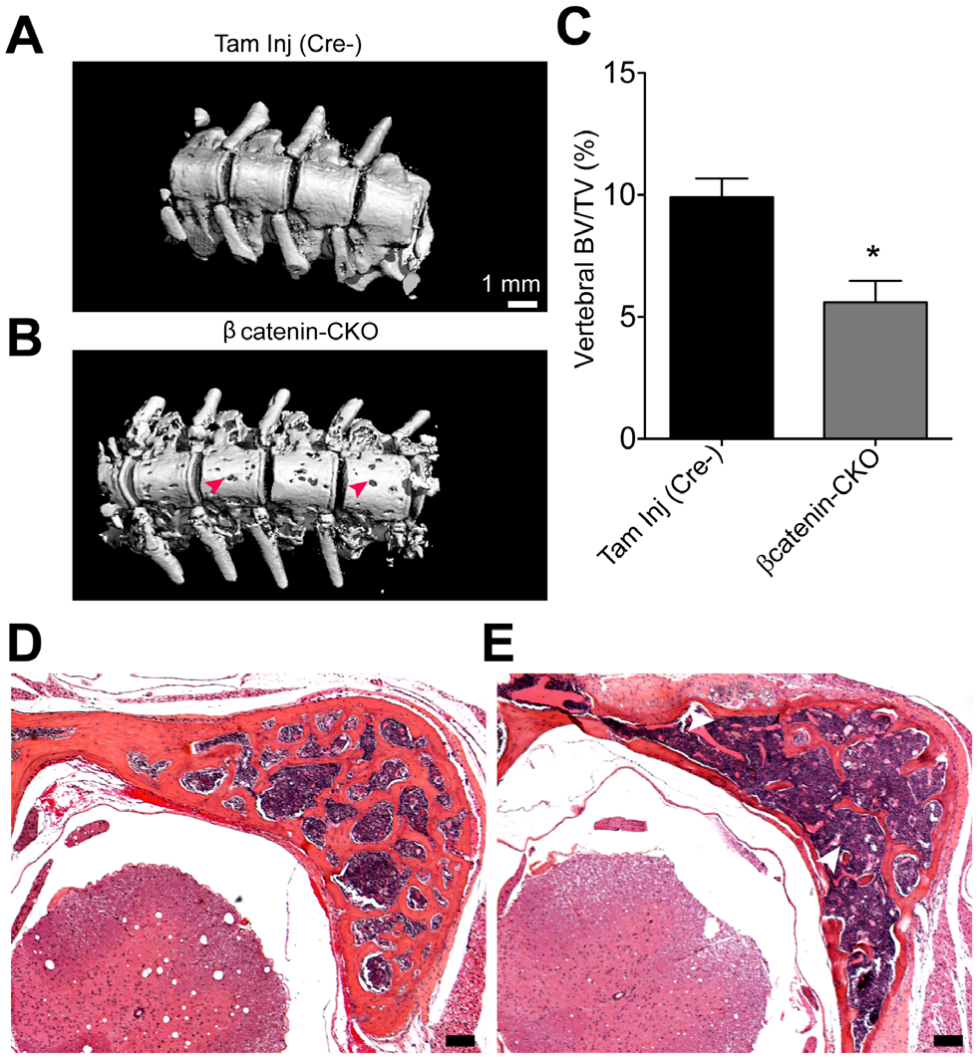
Changes in vertebral bone in βcatenin-CKO animals 10 days post tamoxifen. (**A,B**) High resolution micro CT of vertebrae of (**A**) control and (**B**) βcatenin-CKO animals (red arrowheads point to regions of bone loss) (**C**) mean BV/TV (**D,E**) Hematoxylin-eosin staining of sections of vertebrae of (**D**) control and (**E**) βcatenin-CKO animals (white arrowheads point to loss of trabecular bone) (mean±S.E.M.; n = 3 animals/group; *p<0.05, BV/TV: trabecular bone volume/total volume). (Scale bar: 100 µm).

**Figure 4:**
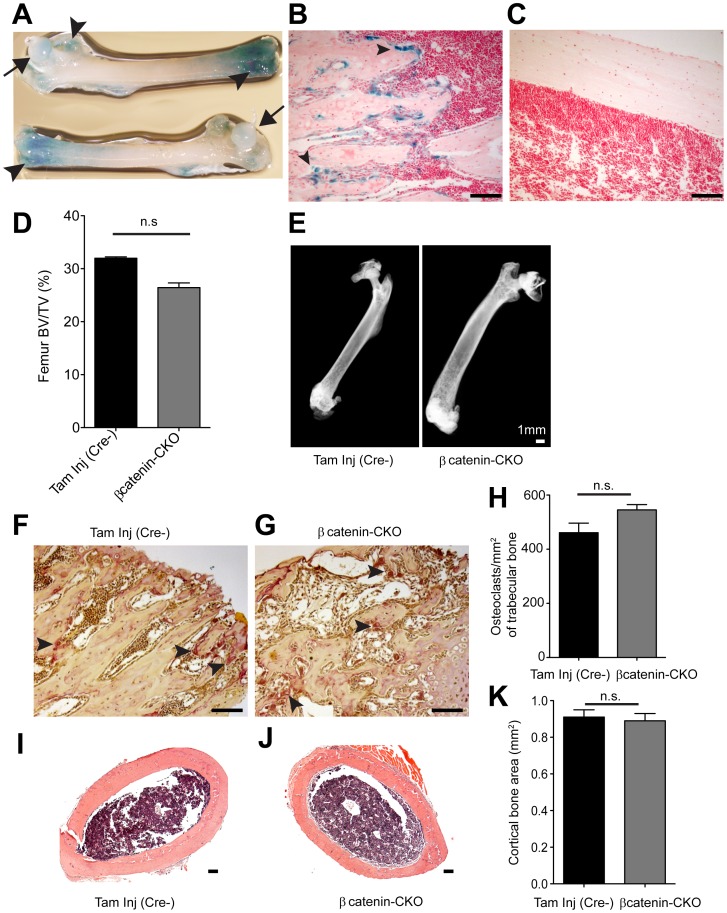
Changes in femur of βcatenin-CKO animals 10 days post tamoxifen. (**A**) X gal staining *in situ* of femur of *Col1a2Cre:R26R^lacZ^* mice (arrowheads point to lacZ expressing regions, arrow shows femoral head) (**B, C**) X gal staining of section of (**B**) metaphyseal end of femur and (**C**) femoral diaphysis (arrowheads point to lacZ expressing cells, with very few cells expressing lacZ in the diaphysis). (**D**) Change in femoral BV/TV in βcatenin-CKO animal (mean±S.E.M, n = 3 animals/group, p>0.05, bone volume measured at metaphyseal ends, BV/TV: trabecular bone volume/total volume) (**E**) Micro CT of femurs and (**F, G**) TRAP staining of metaphyseal region of femurs of (**F**) control and (**G**) βcatenin-CKO animals (arrowheads show TRAP positive cells) and (**H**) number of osteoclasts/mm^2^ of femoral metaphysis (n = 3 femurs/group, n.s = not significant) (**I, J**) Hematoxylin-eosin staining of femoral diaphysis sections of (**I**) control and (**J**) βcatenin-CKO animals and (**K**) histomorphometric assessment of cortical bone area of femoral diaphysis (n = 9 femurs/group, n.s = not significant) (Scale bar: 100 µm).

We next investigated changes in bone density elsewhere in the skeleton. Micro CT of the vertebrae showed extensive loss of vertebral bone with likely loss of both cortical and trabecular bone **(** **Figure 3A,B** **)**. Affected animals demonstrated a 40% significant decrease in vertebral trabecular bone volume (BV/TV), compared to control animals **(** **Figure 3C** **)**. Consistent with observations made on CT scan, hematoxylin-eosin staining showed loss of bony trabeculae **(** **Figure 3D,E** **)**. Xgal staining in situ of long bones such as the femur of the *Col1a2Cre* lineage reporter mice showed expression at the trabecular ends of the long bones **(** **Figure 4A–C** **)** in contrast to the uniform expression across the entire surface of the ribs. Consistent with this expression pattern, there was <10% decrease (p>0.05) in bony volume (BV/TV) of the femoral metaphysis **(** **Figure 4D** **)** in the βcatenin CKO mice. Micro CT of the femur did not reveal any fractures (**Figure 4E**) and TRAP staining demonstrated no significant differences in the number of osteoclasts in the trabecular metaphysis between the βcatenin-CKO animals and control groups **(** **Figure 4F–H** **)**. Histomorphometry of the femoral diaphysis also did not show any significant change in cortical bone **(** **Figure 4I–K** **)**. Increased expression of *Col1a2* in the ribs compared to the femur could potentially explain the relative sparing of bone in the femur.

### Rib Fractures and Death are Ameliorated by Dexamethasone

**Figure 5:**
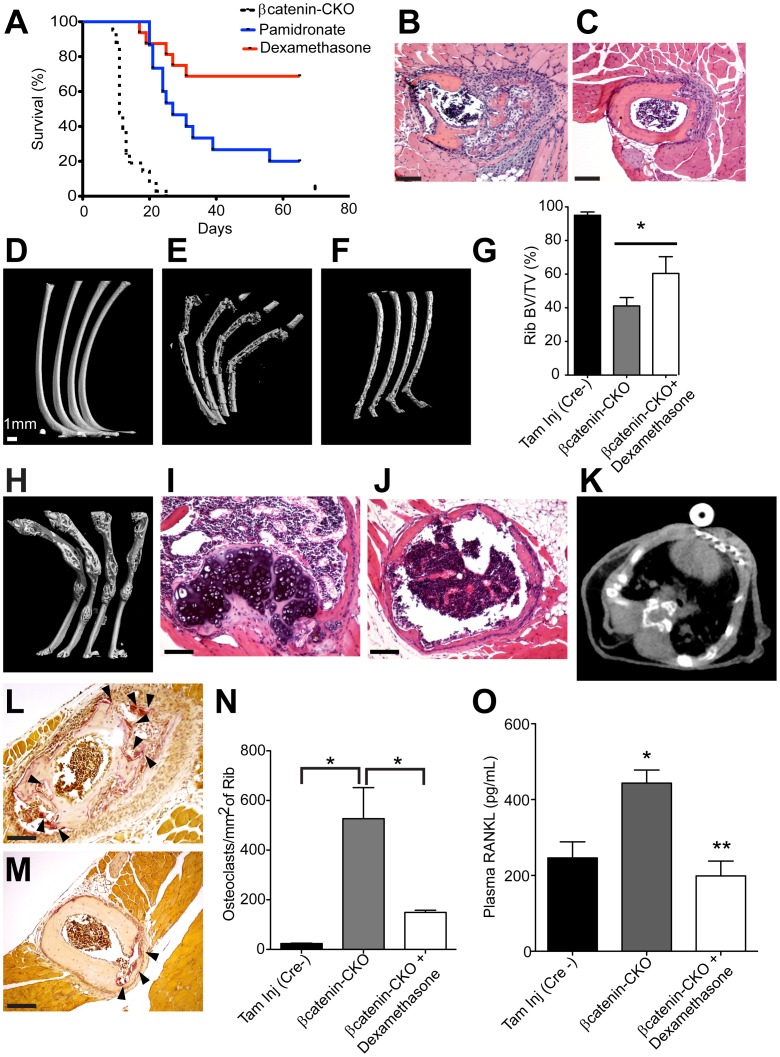
Dexamethasone improves survival and preserves rib structure by decreasing RANKL and osteoclastogenesis in βcatenin-CKO mice. (**A**) Kaplan-Meier survival curve of βcatenin-CKO animals treated with dexamethasone or pamidronate (p<0.005 between dexamethasone and βcatenin-CKO groups, p<0.05 between dexamethasone and pamidronate groups, n = 16 animals in dexamethasone and pamidronate groups) (**B,C**) Hematoxylin-eosin staining of βcatenin-CKO animals 10 days post tamoxifen (**B**) untreated or (**C**) treated with dexamethasone (**D–F**) High resolution micro CT of ribs from (D) Cre negative control animal (**E**) βcatenin-CKO and (**F**) dexamethasone treated βcatenin-CKO 10 days post tamoxifen (**G**) Rib BV/TV 10 days post tamoxifen (mean±S.E.M.; *p<0.05, n = 4 ribs/animal with 3 animals/group) (**H**) Micro CT of ribs of dexamethasone treated βcatenin-CKO 65 days post tamoxifen (**I,J**) Hematoxylin-eosin staining of ribs 65 days post tamoxifen in dexamethasone treated βcatenin-CKO animal showing (**I**) cartilage formation and (**J**) restoration of bone circumference. (**K**) CT scan 65 days post tamoxifen of dexamethasone treated animal showing bilaterally inflated lungs. (**L–N**) TRAP staining 10 days post tamoxifen showing osteoclasts (arrowheads) in ribs of (**L**) untreated and (**M**) dexamethasone treated βcatenin-CKO animals with (**N**) number of TRAP positive cells normalized to rib surface area (mean±S.E.M.; *p<0.05, n = 16 ribs/animal with 3 animals/group) (**O**) Serum RANKL levels 8 days post tamoxifen in control and βcatenin-CKO mice with/without dexamethasone treatment (mean±S.E.M.; *p<0.05 versus Tam Inj Cre-, **p<0.05 versus βcatenin-CKO, n = 10 animals/group). (Scale bar: 100 µm).

We investigated whether inhibition of osteoclast activity would prevent bone resorption and rescue the βcatenin-CKO phenotype. The bisphosphonate, pamidronate (5mg/kg) [22] was administered concurrently with tamoxifen and continued throughout the remaining duration of the experiment. This dose of pamidronate is considered to be a “high dose” and can be effectively used in rapid bone loss such as hypercalcemia of malignancy [23]. Pamidronate at this dose delayed but did not prevent death in the large majority of the animals with only 20% of the animals surviving 65 days after the last dose of tamoxifen **(** **Figure 5A** **)**. This suggested that osteoclastogenesis arising from βcatenin deletion in rib osteoblasts was significantly more aggressive than that seen in bisphosphonate amenable osteoporosis. Osteoblasts regulate osteoclast formation and express the osteoclastogenic cytokine RANKL that leads to increased osteoclast recruitment [8]. Bisphosphonates do not work through regulation of RANKL [23], [24] but glucocorticoids have been shown to decrease RANKL expressed by inflammatory cells in immune mediated arthritis [25]. Although the affected ribs did not contain activated T, B cells or macrophages as shown by the absence of CD8, B220 and F4/80 antigens **(Figure S5C–G)**, given the rapidity of the process we elected to treat the βcatenin-CKO mice with this immune modulator. Dexamethasone (dexamethasone phosphate equivalent to 1mg/kg of dexamethasone) was started concurrently with tamoxifen injections and continued thrice weekly throughout the duration of survival analysis (65 days post cessation of tamoxifen). Strikingly, 75% of the dexamethasone treated βcatenin-CKO animals survived at 65 days compared to 0% survival at day 25 in the tamoxifen only group (p<0.0001) (**Figure 5A**). No gender specific differences were observed with equal numbers of males and females surviving. Hematoxylin-eosin staining of rib sections harvested 10 days following cessation of tamoxifen demonstrated greater amount of bone in the dexamethasone treated animals **(** **Figure 5B,C** **)**. Consistent with histological assessment, high resolution micro CT scan of the ribs at 10 days post tamoxifen showed 46% increase in rib BV/TV of the ribs of dexamethasone treated animals (**Figure 5D–G**) (p<0.05, n = 3 animals/group with 4 ribs examined/animal). At 65 days post tamoxifen, the ribs in the surviving animals showed extensive bony remodeling **(** **Figure 5H** **)**. Hematoxylin-eosin staining of ribs 65 days post tamoxifen in dexamethasone treated animals showed evidence of healing by endochondral ossification with cartilage formation at the surface of the ribs and partial restoration of the bony circumference **(** **Figure 5I,J** **)**. Importantly, CT scan of the chest in dexamethasone treated βcatenin-CKO animals 65 days post-tamoxifen demonstrated bilaterally inflated lungs without evidence of lung collapse **(** **Figure 5K** **)**.

TRAP staining performed 10 days post tamoxifen showed 8 fold increase in osteoclasts in the ribs of βcatenin-CKO but 60% reduction in the number of osteoclasts in ribs of animals treated with dexamethasone **(** **Figure 5L–N** **)**, demonstrating that dexamethasone was significantly attenuating osteoclastogenesis. Measurement of serum RANKL 8 days post tamoxifen dosing demonstrated a significant elevation in βcatenin-CKO animals compared to control mice (Tamoxifen injected Cre negative) (443±34 pg/mL vs 246±42 pg/mL, mean±S.E.M, p<0.05) **(** **Figure 5O** **)**. In animals treated with dexamethasone, serum RANKL levels (199±39 pg/mL) were indistinguishable from control animals **(** **Figure 5O** **)**. Immunohistochemistry for RANKL on frozen sections of ribs showed abundant RANKL expression associated with the rib osteoblast infiltrate in the βcatenin-CKO animals **(Figure S7A,B)**. In contrast, concurrent dexamethasone decreased RANKL expression mirroring the decrease in serum RANKL **(Figure S7C)**.

We confirmed that the bone sparing effects of dexamethasone were not a result of dexamethasone induced suppression of *Col1a2* driven Cre-recombinase. In βcatenin-CKO:R26R^lacZ^ animals, where Cre expressing cells are identified by lacZ expression, dexamethasone treatment did not decrease the number of lacZ expressing cells at 11 days following initiation of dexamethasone as compared to βcatenin-CKO:R26R^lacZ^ animals which were not treated with dexamethasone **(Figure S8)**. Furthermore, initiating dexamethasone after completing tamoxifen treatment still led to significantly higher survival rates, albeit less than when dexamethasone was started concurrent with tamoxifen injections **(Figure S9)**. Taken together, these data suggest that dexamethasone, particularly if initiated early has remarkable bone preserving effects during a state of rapid and aggressive osteoclastogenesis.

## Discussion

Our study demonstrates a remarkable phenotype of aggressive osteoclastogenesis of the thoracic cage within days of osteoblast specific deletion of βcatenin that results in a disappearing thoracic skeleton incapable of supporting ventilation and life. Our findings highlight the role of *Col1a2* osteoblast βcatenin in determining rib fragility. Previous studies with embryonic deletion of βcatenin in *Col1a1* expressing osteoblasts did not cause such a dramatic phenotype and there was no note of rib lesions leading to increased mortality [8]. These differences may be secondary to different cell specific promoters driving *Cre* recombinase or other compensatory pathways preventing or rescuing such phenotypes during embryonic development. The rate of cortical bone turnover is highly site-specific and our data suggests that rib cortical bone has high turnover rates. Site specific bone resorption might depend on pathological RANKL expression by local osteoblasts, rather than by the secretion of RANKL by matrix embedded bone osteocytes thought to be predominant during unloading-associated osteoclastogenesis in trabecular bone [26] or in remodeling associated with normal skeletal development [27]. Indeed, our results show that βcatenin deletion in rib cortical osteoblasts leads to unusually high local expression of RANKL demonstrated histologically. This might suggest that RANKL is expressed by osteoblasts under pathological conditions, and lead to site specific bone resorption, similar to the expression of RANKL by T-lymphocytes after ovariectomy [28] or from multiple sources in inflamed joints [29], [30], [31].

Our study suggests that corticosteroids might, used judiciously, limit pathological osteoclastogenesis. This is surprising, as glucocorticoids have been shown to enhance the RANKL axis in osteoblasts *in vitro* and chronic glucocorticoid therapy is associated with bone loss and fractures [16], [32]. However, glucocorticoid injection into joints decreases RANKL expression by synovial fluid lymphocytes, and, in TNF primed osteoblasts, downregulates RANKL/osteoprotegerin expression [25]. Moreover, when inflammation is ameliorated in inflammatory arthritis, bone formation is induced, in part through improvement in Wnt antagonism [33]. It is possible that the intense osteoclastogenesis consequent to βcatenin deletion in osteoblasts in adults in our model is accelerated by inflammatory signals. Although we did not find evidence of lymphocytic or macrophage infiltration in resorbing ribs, it is possible that osteoblasts themselves might be sources of corticosteroid-responsive cytokines.

Aggressive osteoclastogenesis in the ribs and at other skeletal sites can be observed in metastatic cancers and multiple myeloma [12], [34]. Future studies can capitalize on our findings to uncover interactions that regulate bone destruction, in these conditions, and may be improved by stimulating βcatenin signaling. Further, the model of accelerated osteoclastogenesis and aggressive bone turnover reported here presents a vehicle for rapid pharmacologic testing of anti-resorptive and other bone sparing agents. In summary, we present here a novel model of osteoclastogenesis with a predominant rib phenotype and demonstrate that glucocorticoids, through decreasing RANKL and limiting osteoclastogenesis, may have a role in the treatment of bone loss from rapid osteoclastogenesis.

## Supporting Information

Figure S1 **Distribution of** ***Col1a2*** **expressing cells in different organs.** Xgal staining of sections of different organs harvested from adult *Col1a2CreERT:R26R^lacz^* mice following induction of Cre shows lacZ expressing cells (arrowheads) in (**A**) coronary artery (**B**) cardiac muscle interstitium (**C**) splenic capsule (**D**) glomerular mesangium (**E**) lung (**F**) liver (**G**) skeletal muscle interstitium (**H**) bone. (Scale bar: 100 µm).
(TIF)

Figure S2 **Pulmonary function in βcatenin-CKO mice 10 days post tamoxifen.** Invasive measurements of pulmonary ventilation. (mean±S.E.M., *p<0.05 compared to control groups, n = 6 animals/group).
(TIF)

Figure S3 **White blood cell and differential counts in βcatenin-CKO mice 10 days post tamoxifen.** Peripheral white blood cell counts and differential counts determined by automated analyzer. (mean±S.E.M., *p<0.05 compared to other control groups, n = 10 animals/group, blue line refers to lower limit of normal counts).
(TIF)

Figure S4 **Deletion of βcatenin in** ***Col1a2*** **expressing cells of βcatenin-CKO mice.** (**A**) Immunofluorescent staining for osteoblast (alkaline phosphatase, red) and βgalactosidase (green) in *Col1a2CreERT:R26R^lacZ^* mice demonstrates labeling of osteoblasts (arrowheads) (**B**) Immunofluorescent staining for βcatenin (green) and alkaline phosphatase (red) in βcatenin-CKO mice demonstrates absence of βcatenin in osteoblasts (arrows). (Arrowheads point to presence of βcatenin expressing osteoblasts at the margin of the bone). (End:endosteal surface; Per:Periosteal surface; Scale bar: 50 µm).
(TIF)

Figure S5 **Cellular infiltrate associated with rib destruction consists of osteoblasts.** (**A,B**) Alkaline phosphatase staining of (**A**) control and (**B**) βcatenin CKO animal, 10 days post tamoxifen demonstrates osteoblasts (arrowheads) around bony circumference of the rib. (*points to bony rib in control and destroyed rib in βcatenin-CKO animal) (**C-E**) Immunohistochemistry on frozen rib sections of βcatenin-CKO animal for (**C**) B220 (**D**) CD8 and (**E**) F4/80 antigens shows absence of staining in the infiltrate surrounding the destroyed ribs (**F,G**) Immunohistochemistry for (**F**) B220 and (**G**) F4/80 on unrelated sections with known expression of these markers (red arrowheads; positive controls). (Scale bar: 100 µm).
(TIF)

Figure S6 **Osteoid and calcium loss in ribs of βcatenin-CKO animals 10 days post tamoxifen.** (**A, B**) Masson-trichrome staining of ribs of (**A**) control and (**B**) βcatenin-CKO animal (collagen matrix is stained blue) and (**C**) quantitation of cortical rib surface area (n = 16 ribs/animal with 3 animals/group) (**D, E**) Von Kossa staining of frozen sections of ribs from (**D**) control and (**E**) βcatenin-CKO animal (calcium is stained black) and (**F**) quantitation of surface area staining for Von Kossa (n = 9 ribs) (mean±S.E.M.; *p<0.05, Scale bar: 100 µm).
(TIF)

Figure S7 **RANKL expression in ribs of βcatenin-CKO and control animals.** RANKL expression (arrowheads), 10 days post tamoxifen, in ribs of (**A**) Cre negative (**B**) βcatenin-CKO and (**C**) dexamethasone treated βcatenin-CKO mice. (Scale bar: 100 µm).
(TIF)

Figure S8 **Dexamethasone does not suppress Cre recombinase in βcatenin-CKO animals.** βcatenin-CKO:R26RlacZ animals were (A) untreated or (B) treated with dexamethasone administered concomitantly with tamoxifen thrice weekly as described. Animals were harvested and ribs examined for the presence of lac Z expressing cells 11 days following completion of tamoxifen injections (arrows).
(TIF)

Figure S9 **Survival curve of βcatenin-CKO mice following dexamethasone injections initiated after completion of tamoxifen injections.** βcatenin-CKO animals were injected with tamoxifen for 10 days and then untreated (red) or treated with dexamethasone (green) (1mg/kg thrice weekly). (p<0.0001 between untreated and dexamethasone treated groups).
(TIF)

Table S1 **Echocardiographic parameters of cardiac function in βcatenin-CKO and control mice before injection and 10 days post cessation of tamoxifen or oil injection.** LVEDD, left ventricular end-diastolic dimension; LVESD, left ventricular end-systolic dimension; FS, fractional shortening, calculated as (LVEDD-LVESD)/LVEDD×100; EF%, ejection fraction calculated as (End diastolic volume-End systolic volume)/End diastolic volume×100. Data expressed as mean±S.D. None of the cardiac parameters were statistically significantly different between the βcatenin-CKO mice and other groups either prior to post injection (2 ways Anova).
(TIF)

Table S2 **Plasma chemistry in βcatenin-CKO and control mice 10 days following cessation of tamoxifen.** NS = not significant. n = 10 animals/group, mean±S.E.M.
(TIF)

Table S3 **Red Blood Cell and Platelet Counts in βcatenin-CKO and control mice 10 days following tamoxifen.** HCT: hematocrit; MCV: mean corpuscular volume; RBC: Red blood cell; HGB: Hemoglobin; MCH: mean corpuscular hemoglobin; MCHC: mean corpuscular hemoglobin concentration; RDW: red blood cell distribution width; MPV: mean platelet volume (n = 10 animals/group, mean± S.E.M., measurements made in triplicate, NS = not significant *p<0.05 versus other groups, one way Anova).
(TIF)

Video S1 **Three-dimensional CT scan reconstruction of rib cage of Cre negative control animal 10 days post tamoxifen.** (Orientation: A = Anterior, S = Superior, P = Posterior, I = Inferior, R = Right, L = Left).
(MOV)

Video S2 **Three-dimensional CT scan reconstruction of rib cage of βcatenin-CKO animal 10 days post tamoxifen.** (Orientation: A = Anterior, S = Superior, P = Posterior, I = Inferior, R = Right, L = Left).
(MOV)
