# Supplementary figures and images for: Rib Fractures and Death from Deletion of Osteoblast βcatenin in Adult Mice Is Rescued by Corticosteroids
Source: PLoS One. 2013 Feb 5;8(2):e55757. doi: 10.1371/journal.pone.0055757 (PMC3564851; doi:10.1371/journal.pone.0055757)

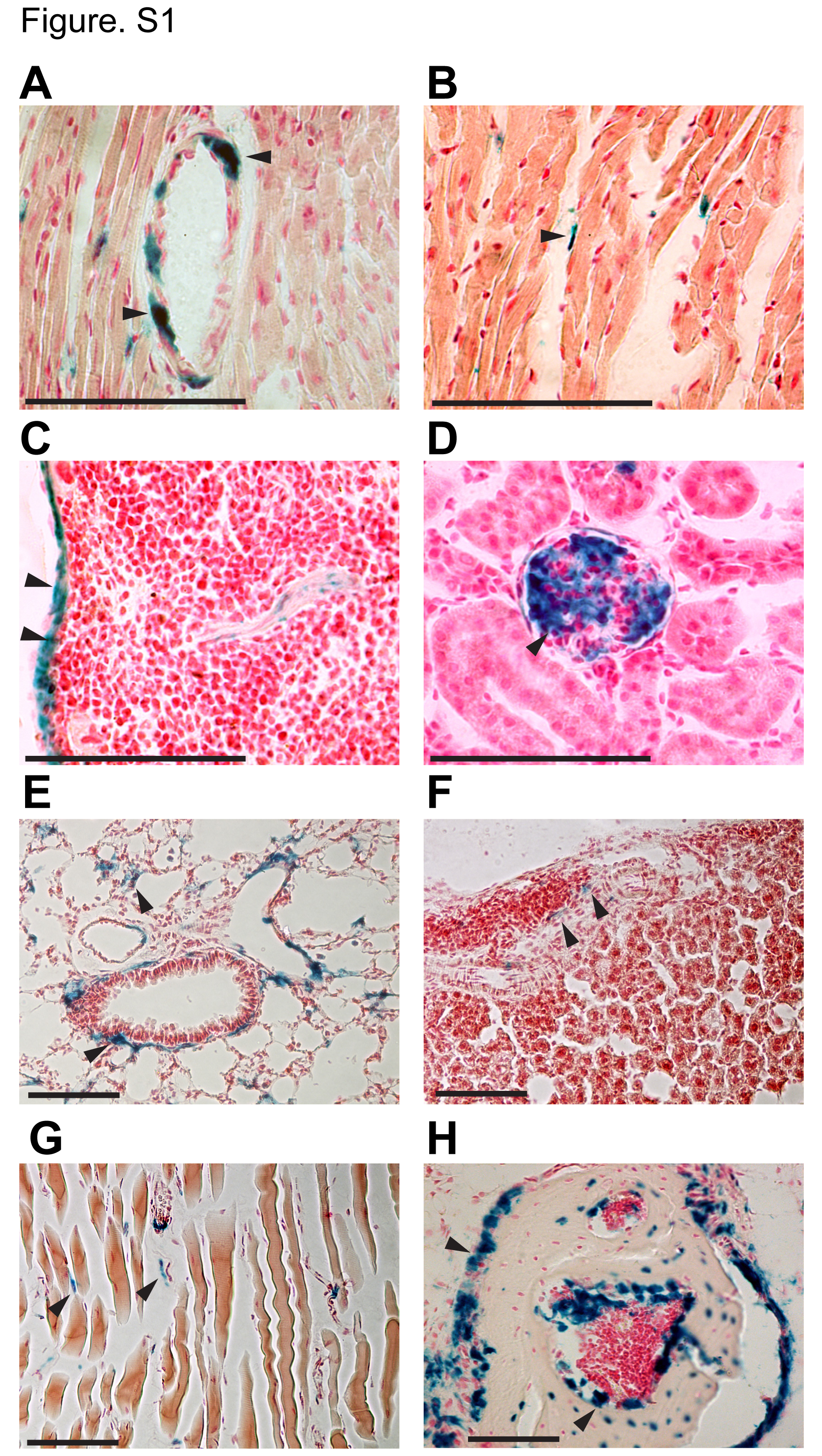

Supplement: Figure S1 — Distribution of Col1a2 expressing cells in different organs. Xgal staining of sections of different organs harvested from adult Col1a2CreERT:R26Rlacz mice following induction of Cre shows lacZ expressing cells (arrowheads) in (A) coronary artery (B) cardiac muscle interstitium (C) splenic capsule (D) glomerular mesangium (E) lung (F) liver (G) skeletal muscle interstitium (H) bone. (Scale bar: 100 µm). (TIF) [file pone.0055757.s001.tif]

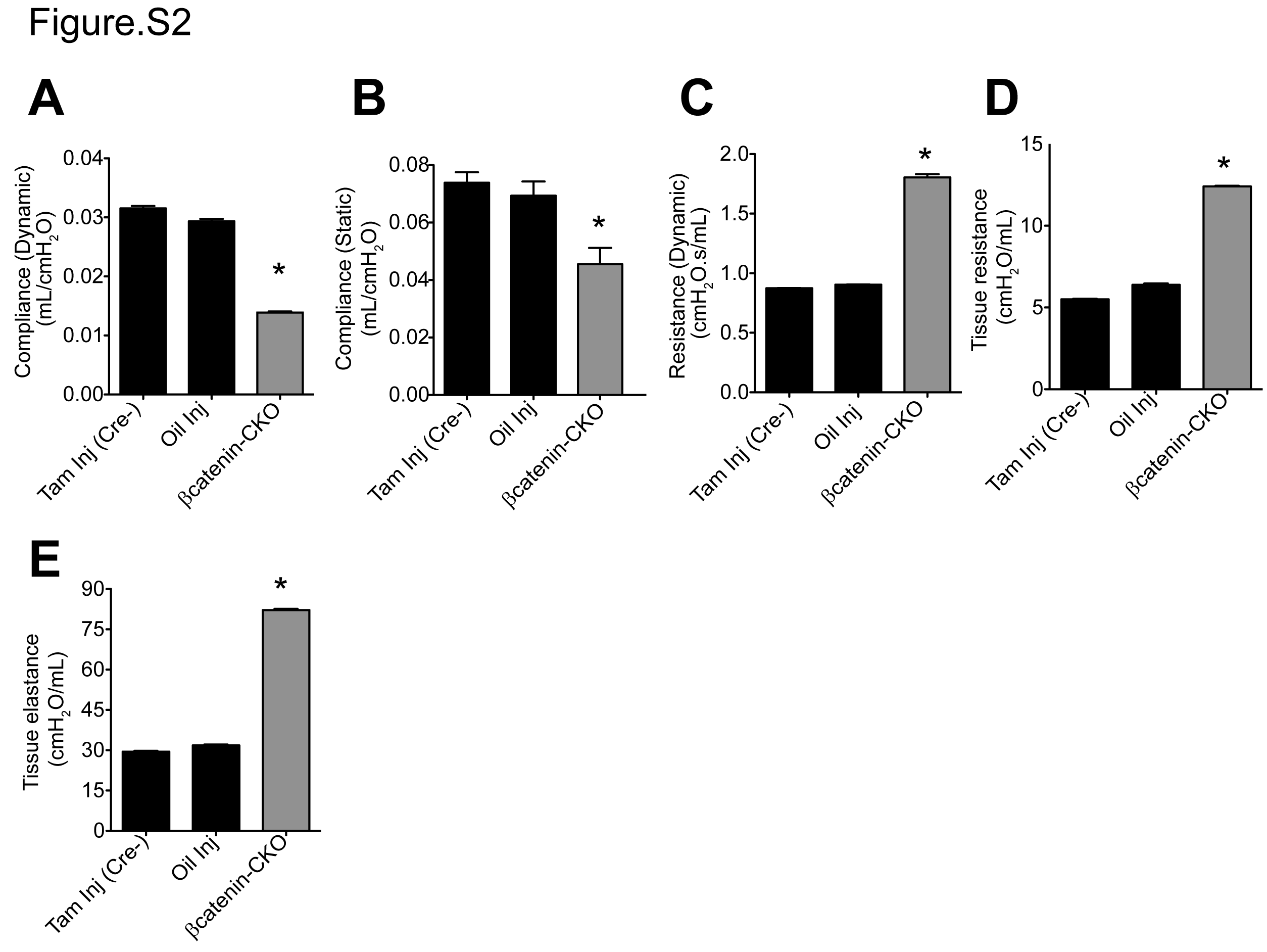

Supplement: Figure S2 — Pulmonary function in βcatenin-CKO mice 10 days post tamoxifen. Invasive measurements of pulmonary ventilation. (mean±S.E.M., *p<0.05 compared to control groups, n = 6 animals/group). (TIF) [file pone.0055757.s002.tif]

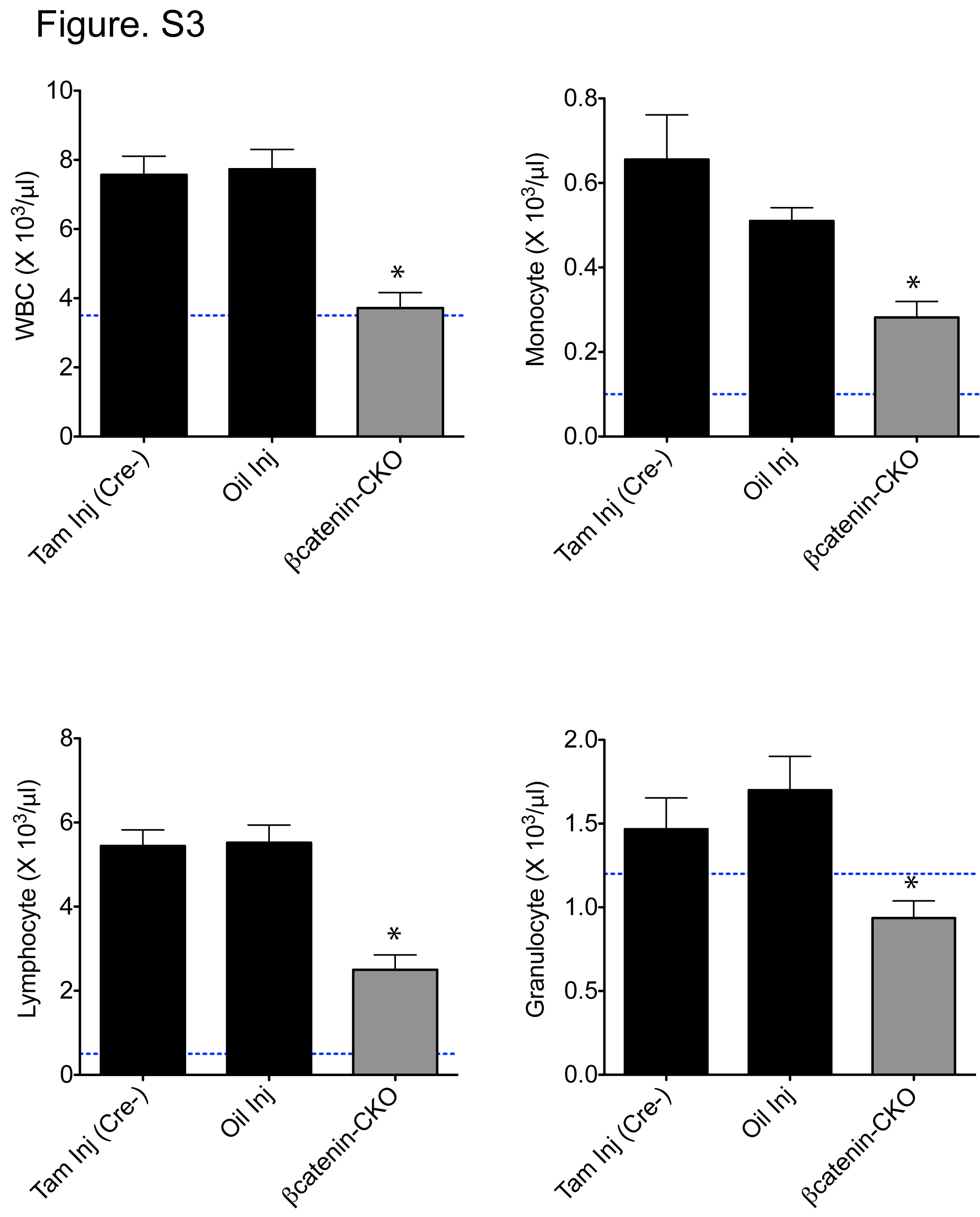

Supplement: Figure S3 — White blood cell and differential counts in βcatenin-CKO mice 10 days post tamoxifen. Peripheral white blood cell counts and differential counts determined by automated analyzer. (mean±S.E.M., *p<0.05 compared to other control groups, n = 10 animals/group, blue line refers to lower limit of normal counts). (TIF) [file pone.0055757.s003.tif]

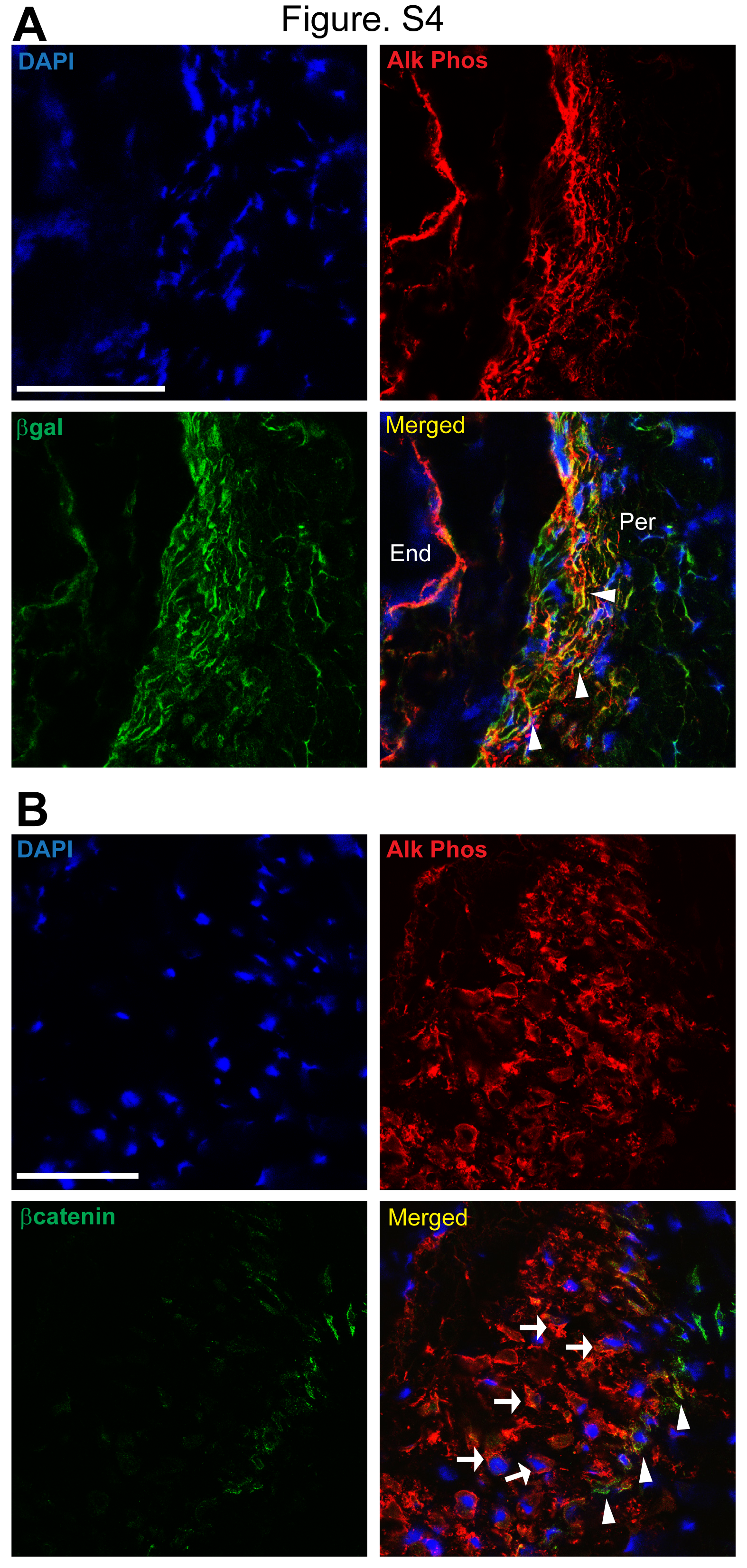

Supplement: Figure S4 — Deletion of βcatenin in Col1a2 expressing cells of βcatenin-CKO mice. (A) Immunofluorescent staining for osteoblast (alkaline phosphatase, red) and βgalactosidase (green) in Col1a2CreERT:R26RlacZ mice demonstrates labeling of osteoblasts (arrowheads) (B) Immunofluorescent staining for βcatenin (green) and alkaline phosphatase (red) in βcatenin-CKO mice demonstrates absence of βcatenin in osteoblasts (arrows). (Arrowheads point to presence of βcatenin expressing osteoblasts at the margin of the bone). (End:endosteal surface; Per:Periosteal surface; Scale bar: 50 µm). (TIF) [file pone.0055757.s004.tif]

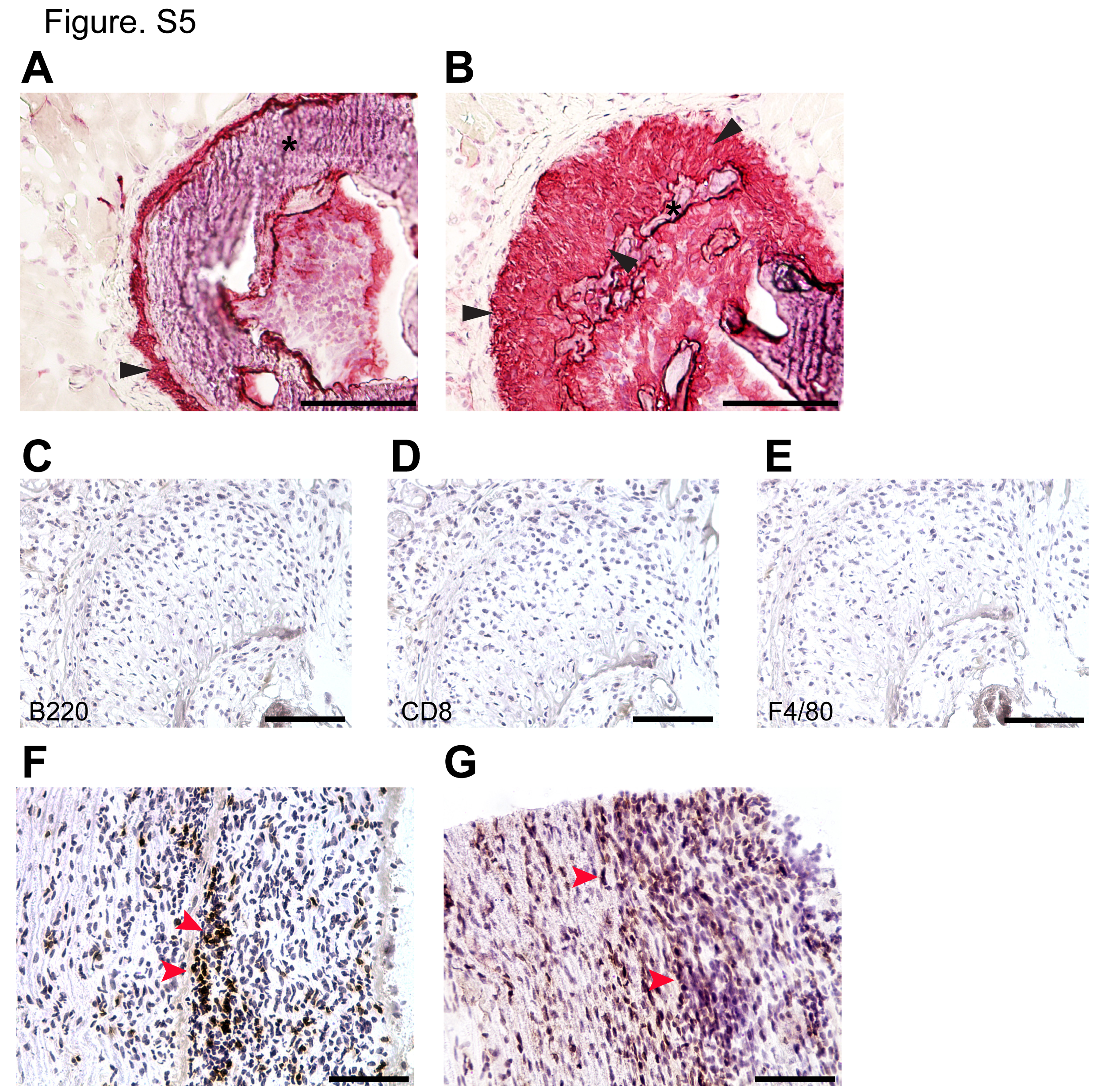

Supplement: Figure S5 — Cellular infiltrate associated with rib destruction consists of osteoblasts. (A,B) Alkaline phosphatase staining of (A) control and (B) βcatenin CKO animal, 10 days post tamoxifen demonstrates osteoblasts (arrowheads) around bony circumference of the rib. (*points to bony rib in control and destroyed rib in βcatenin-CKO animal) (C-E) Immunohistochemistry on frozen rib sections of βcatenin-CKO animal for (C) B220 (D) CD8 and (E) F4/80 antigens shows absence of staining in the infiltrate surrounding the destroyed ribs (F,G) Immunohistochemistry for (F) B220 and (G) F4/80 on unrelated sections with known expression of these markers (red arrowheads; positive controls). (Scale bar: 100 µm). (TIF) [file pone.0055757.s005.tif]

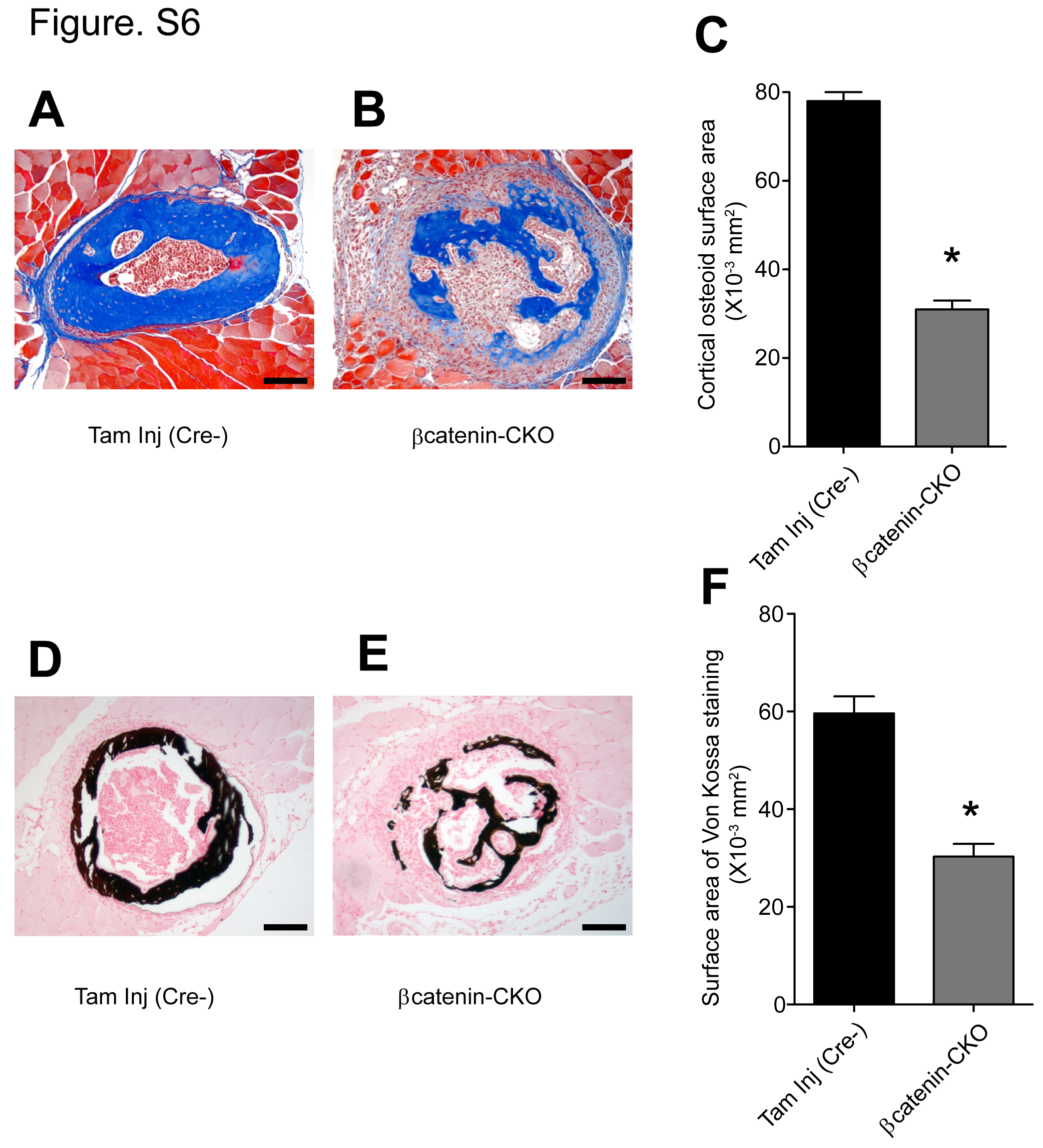

Supplement: Figure S6 — Osteoid and calcium loss in ribs of βcatenin-CKO animals 10 days post tamoxifen. (A, B) Masson-trichrome staining of ribs of (A) control and (B) βcatenin-CKO animal (collagen matrix is stained blue) and (C) quantitation of cortical rib surface area (n = 16 ribs/animal with 3 animals/group) (D, E) Von Kossa staining of frozen sections of ribs from (D) control and (E) βcatenin-CKO animal (calcium is stained black) and (F) quantitation of surface area staining for Von Kossa (n = 9 ribs) (mean±S.E.M.; *p<0.05, Scale bar: 100 µm). (TIF) [file pone.0055757.s006.tif]

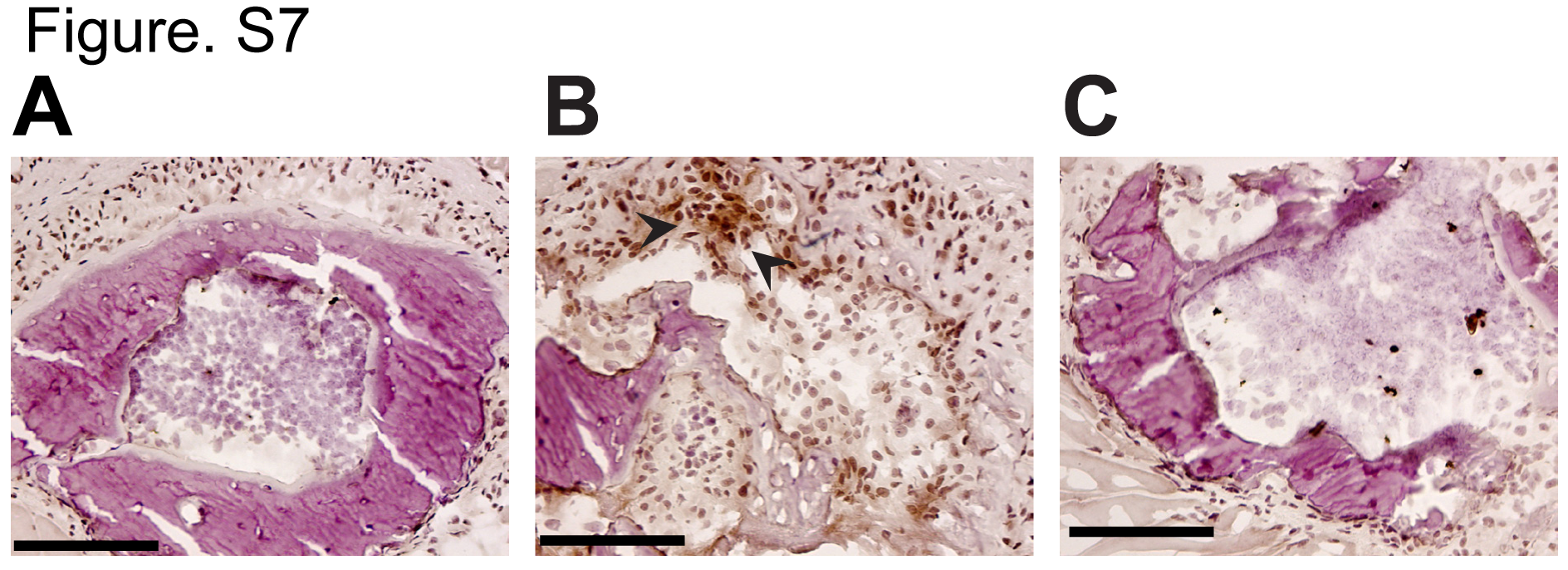

Supplement: Figure S7 — RANKL expression in ribs of βcatenin-CKO and control animals. RANKL expression (arrowheads), 10 days post tamoxifen, in ribs of (A) Cre negative (B) βcatenin-CKO and (C) dexamethasone treated βcatenin-CKO mice. (Scale bar: 100 µm). (TIF) [file pone.0055757.s007.tif]

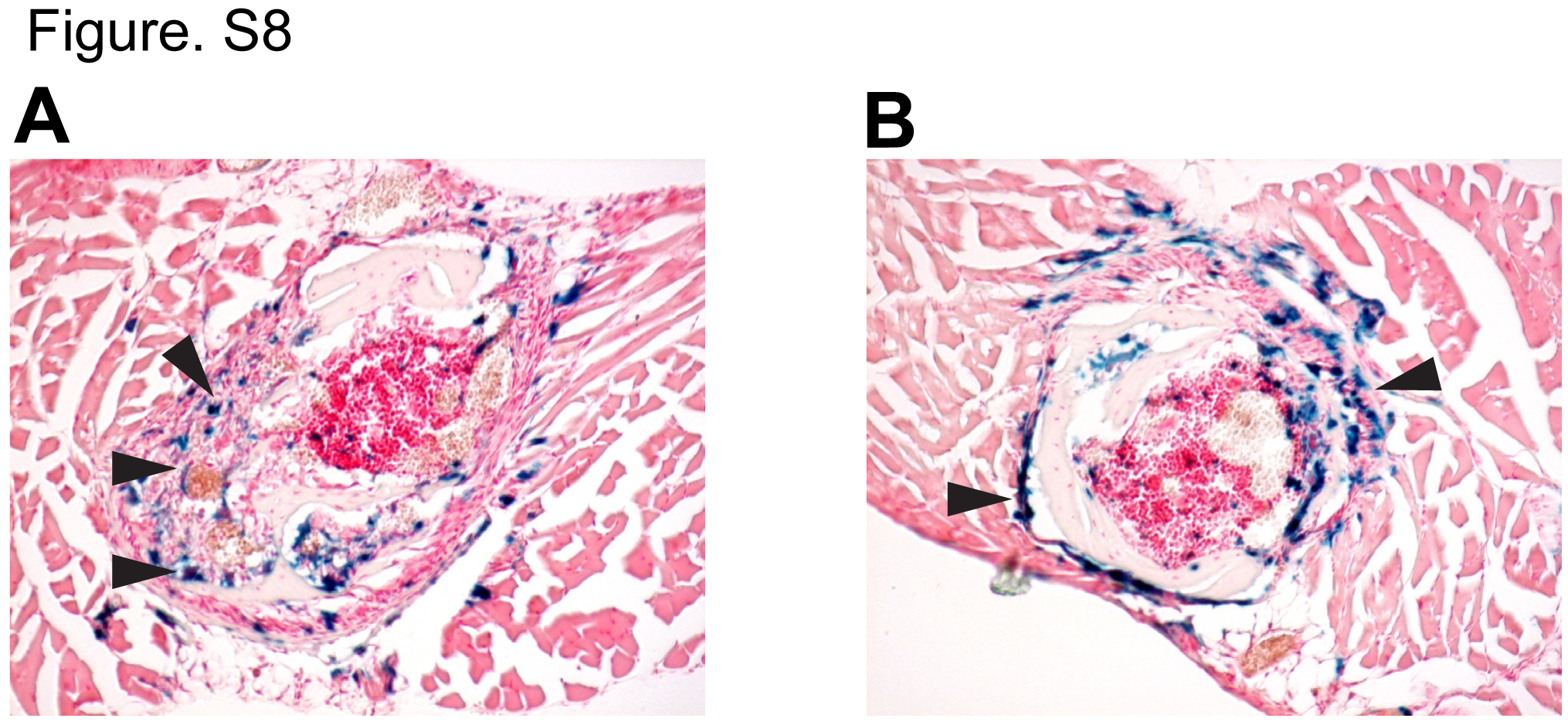

Supplement: Figure S8 — Dexamethasone does not suppress Cre recombinase in βcatenin-CKO animals. βcatenin-CKO:R26RlacZ animals were (A) untreated or (B) treated with dexamethasone administered concomitantly with tamoxifen thrice weekly as described. Animals were harvested and ribs examined for the presence of lac Z expressing cells 11 days following completion of tamoxifen injections (arrows). (TIF) [file pone.0055757.s008.tif]

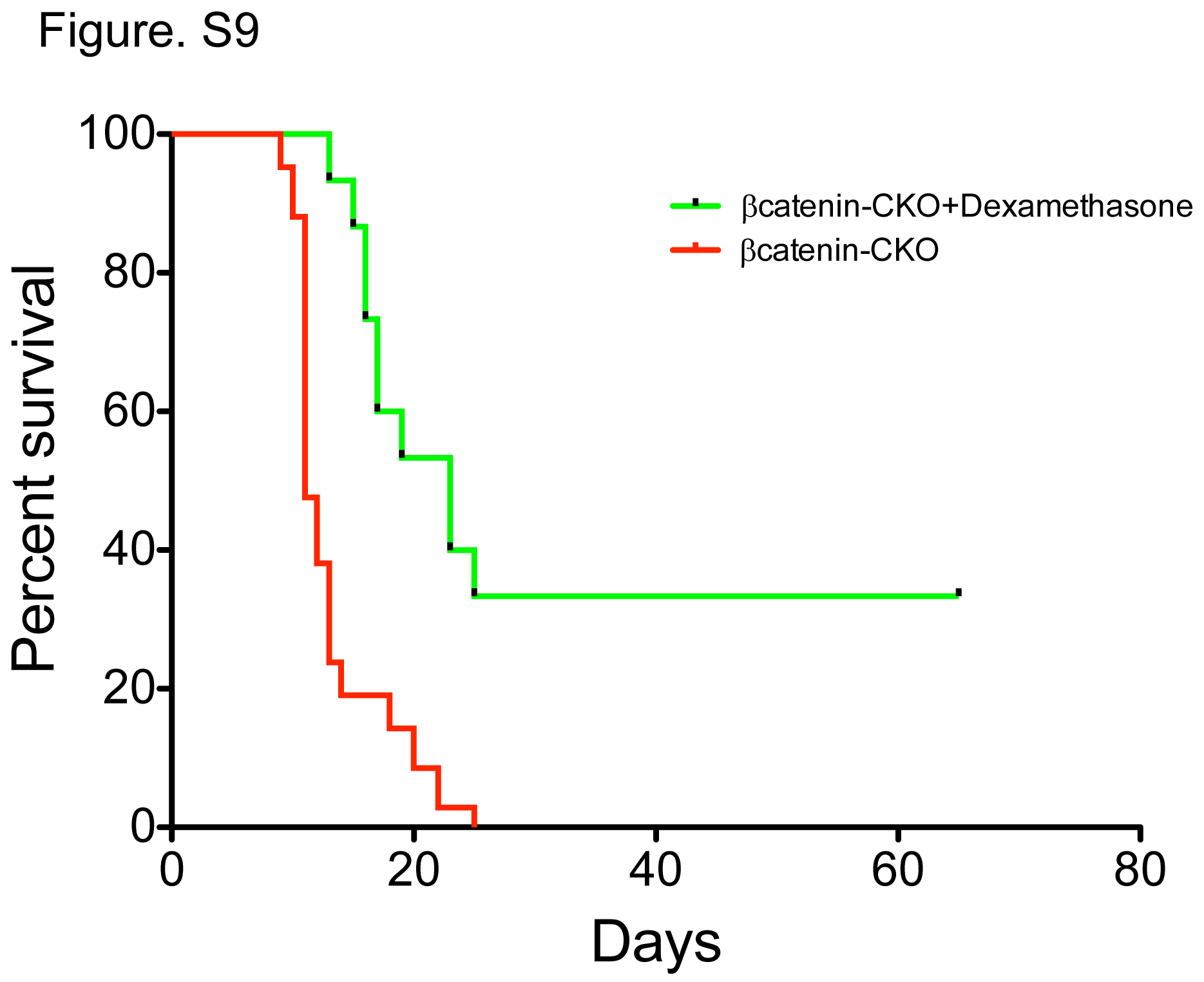

Supplement: Figure S9 — Survival curve of βcatenin-CKO mice following dexamethasone injections initiated after completion of tamoxifen injections. βcatenin-CKO animals were injected with tamoxifen for 10 days and then untreated (red) or treated with dexamethasone (green) (1mg/kg thrice weekly). (p<0.0001 between untreated and dexamethasone treated groups). (TIF) [file pone.0055757.s009.tif]

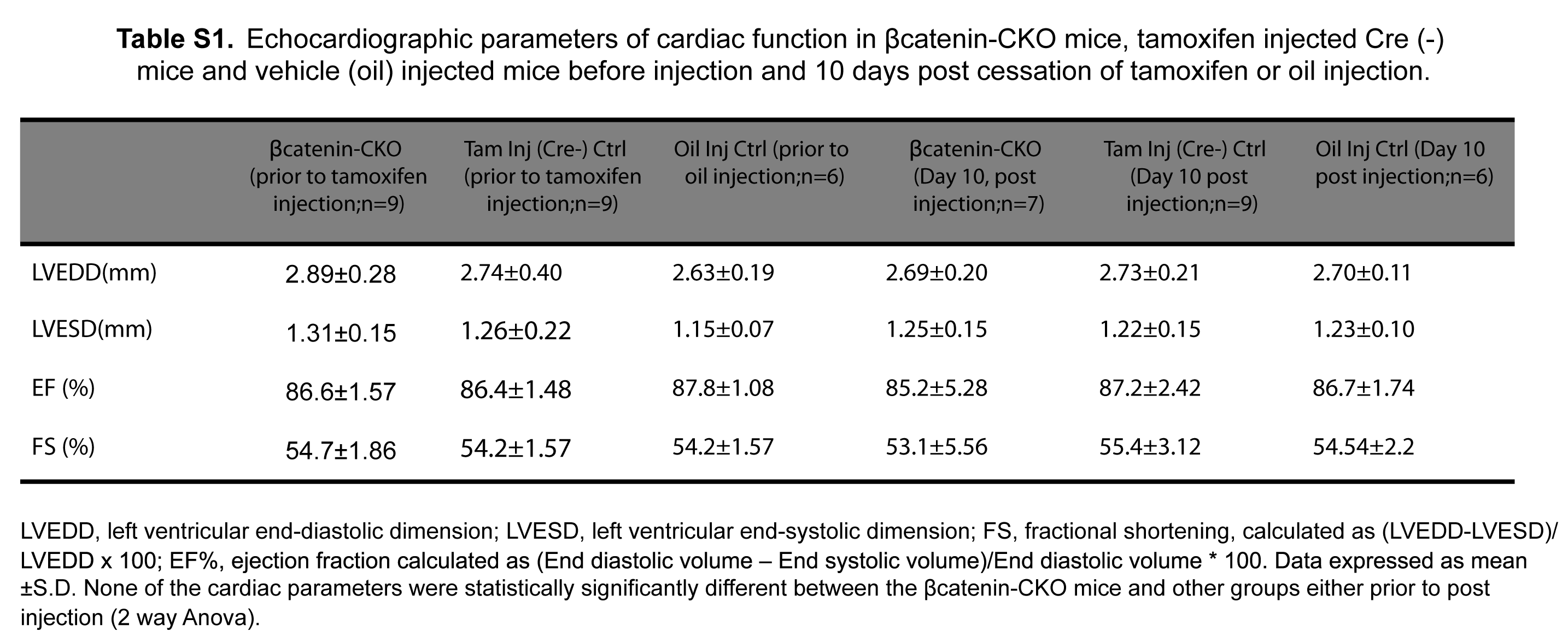

Supplement: Table S1 — Echocardiographic parameters of cardiac function in βcatenin-CKO and control mice before injection and 10 days post cessation of tamoxifen or oil injection. LVEDD, left ventricular end-diastolic dimension; LVESD, left ventricular end-systolic dimension; FS, fractional shortening, calculated as (LVEDD-LVESD)/LVEDD×100; EF%, ejection fraction calculated as (End diastolic volume-End systolic volume)/End diastolic volume×100. Data expressed as mean±S.D. None of the cardiac parameters were statistically significantly different between the βcatenin-CKO mice and other groups either prior to post injection (2 ways Anova). (TIF) [file pone.0055757.s010.tif]

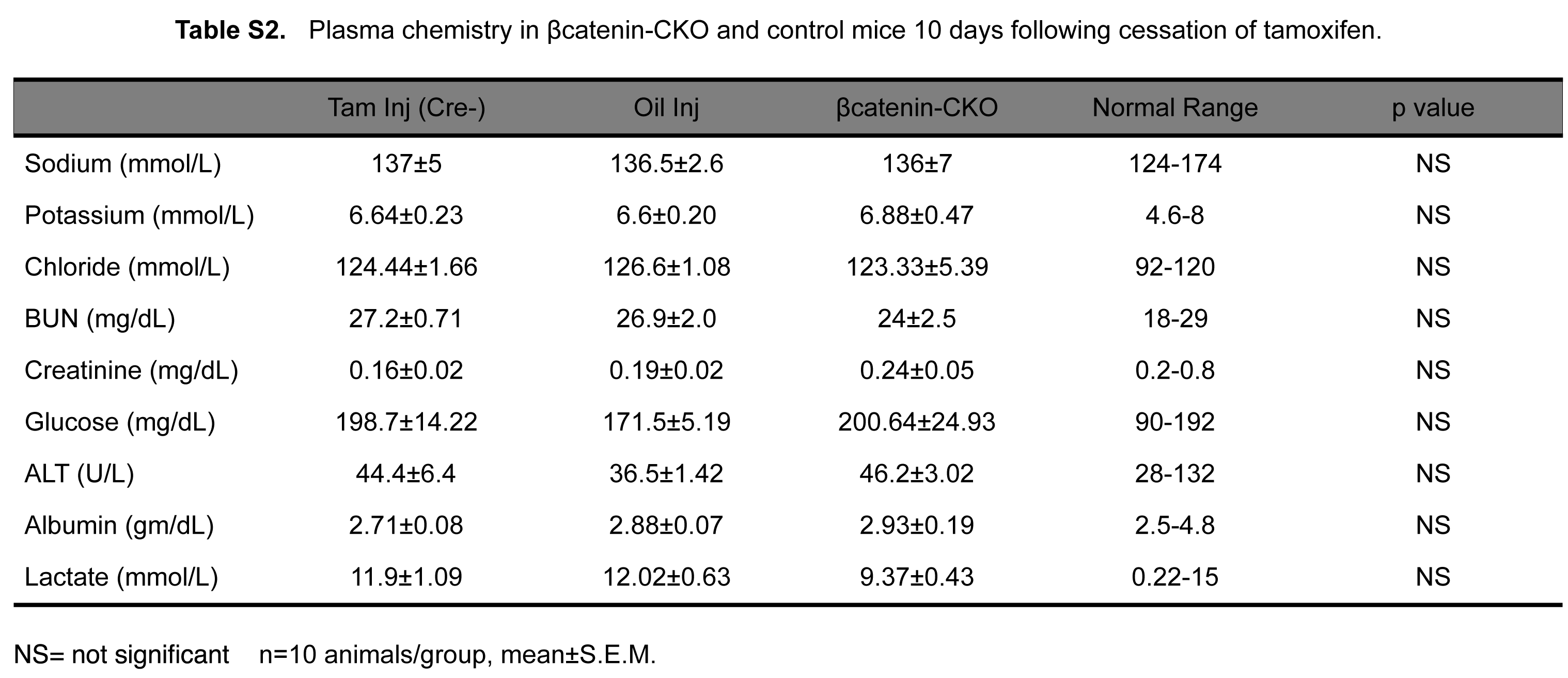

Supplement: Table S2 — Plasma chemistry in βcatenin-CKO and control mice 10 days following cessation of tamoxifen. NS = not significant. n = 10 animals/group, mean±S.E.M. (TIF) [file pone.0055757.s011.tif]

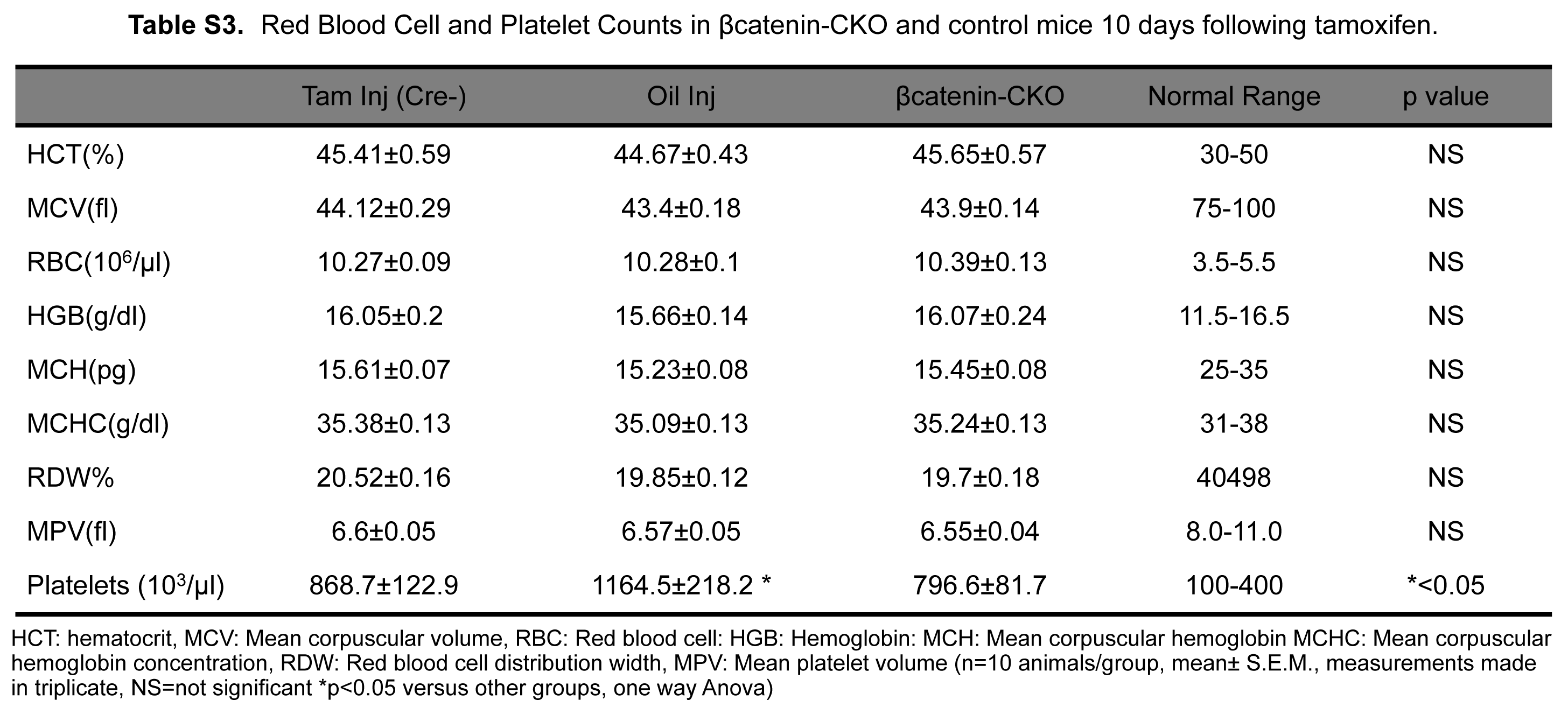

Supplement: Table S3 — Red Blood Cell and Platelet Counts in βcatenin-CKO and control mice 10 days following tamoxifen. HCT: hematocrit; MCV: mean corpuscular volume; RBC: Red blood cell; HGB: Hemoglobin; MCH: mean corpuscular hemoglobin; MCHC: mean corpuscular hemoglobin concentration; RDW: red blood cell distribution width; MPV: mean platelet volume (n = 10 animals/group, mean± S.E.M., measurements made in triplicate, NS = not significant *p<0.05 versus other groups, one way Anova). (TIF) [file pone.0055757.s012.tif]
